# Syndecan-1 (CD138) Modulates Triple-Negative Breast Cancer Stem Cell Properties via Regulation of LRP-6 and IL-6-Mediated STAT3 Signaling

**DOI:** 10.1371/journal.pone.0085737

**Published:** 2013-12-31

**Authors:** Sherif A. Ibrahim, Hebatallah Hassan, Laura Vilardo, Sampath Katakam Kumar, Archana Vijaya Kumar, Reinhard Kelsch, Cornelia Schneider, Ludwig Kiesel, Hans Theodor Eich, Ileana Zucchi, Rolland Reinbold, Burkhard Greve, Martin Götte

**Affiliations:** 1 Department of Gynecology and Obstetrics, University Hospital Münster, Münster, Germany; 2 Institute of Transfusion Medicine and Transplantation Immunology, University Hospital Münster, Münster, Germany; 3 Department of Radiotherapy – Radiooncology, University Hospital Münster, Münster, Germany; 4 ITB-CNR, Segrate Milan, Italy; 5 Department of Zoology, Faculty of Science, Cairo University, Giza, Egypt; University of Patras, Greece

## Abstract

Syndecan-1 (CD138), a heparan sulfate proteoglycan, acts as a coreceptor for growth factors and chemokines and is a molecular marker associated with epithelial-mesenchymal transition during development and carcinogenesis. Resistance of Syndecan-1-deficient mice to experimentally-induced tumorigenesis has been linked to altered Wnt-responsive precursor cell pools, suggesting a potential role of Syndecan-1 in breast cancer cell stem function. However, the precise molecular mechanism is still elusive. Here, we decipher the functional impact of Syndecan-1 knockdown using RNA interference on the breast cancer stem cell phenotype of human triple-negative MDA-MB-231 and hormone receptor-positive MCF-7 cells in vitro employing an analytical flow cytometric approach. Successful Syndecan-1 siRNA knockdown was confirmed by flow cytometry. Side population measurement by Hoechst dye exclusion and Aldehyde dehydrogenase-1 activity revealed that Syndecan-1 knockdown in MDA-MB-231 cells significantly reduced putative cancer stem cell pools by 60% and 27%, respectively, compared to controls. In MCF-7 cells, Syndecan-1 depletion reduced the side population by 40% and Aldehyde dehydrogenase-1 by 50%, repectively. In MDA-MB-231 cells, the CD44(+)CD24(-/low) phenotype decreased significantly by 6% upon siRNA-mediated Syndecan-1 depletion. Intriguingly, IL-6, its receptor sIL-6R, and the chemokine CCL20, implicated in regulating stemness-associated pathways, were downregulated by >40% in Syndecan-1-silenced MDA-MB-231 cells, which showed a dysregulated response to IL-6-induced shifts in E-cadherin and vimentin expression. Furthermore, activation of STAT-3 and NFkB transcription factors and expression of a coreceptor for Wnt signaling, LRP-6, were reduced by >45% in Syndecan-1-depleted cells compared to controls. At the functional level, Syndecan-1 siRNA reduced the formation of spheres and cysts in MCF-7 cells grown in suspension culture. Our study demonstrates the viability of flow cytometric approaches in analyzing cancer stem cell function. As Syndecan-1 modulates the cancer stem cell phenotype via regulation of the Wnt and IL-6/STAT3 signaling pathways, it emerges as a promising novel target for therapeutic approaches.

## Introduction

Breast cancer is a complex disease and is the second leading cause of cancer mortality among women worldwide [1]. Several lines of evidence suggest that, in contrast to the bulk of the tumor, a subset of cancer cells is characterized by the property of self-renewal, unlimited proliferative potential, expression of multidrug-resistance proteins, active DNA repair capacity, apoptosis resistance, and an enormous developmental plasticity [2-7]. Due to these properties, these cancer stem cells (CSCs) display increased resistance to chemo- [8,9] and radiotherapy [10,11] and have the potential to reconstitute the bulk tumor after an otherwise successful therapy [9,12,13]. Furthermore, CSCs have been linked to an increased incidence of distant metastases [14-16]. Thus, targeted therapeutic interventions focused on CSCs might considerably improve cancer therapy [3]. 

Multiparametric flow cytometric approaches play a key role in the analysis of CSC function [3,17,18]. Side population (SP) analysis has been shown to enrich breast CSCs [19]. The SP can be identified by flow cytometry based on their property of effluxing the fluorescent dye Hoechst 33342 via ATP-binding cassette transporter proteins such as ABCG/Brcp1 [3,20,21]. Furthermore, breast CSCs can be isolated based on expression of CD44(+)/CD24(-/low), and aldehyde dehydrogenase activity (ALDH1+) [22,23]. Noteably, CD44(+)/CD24(-/low) breast cancer stem-like cells are associated with tumor recurrence [24] and play a pivotal role in the clinical behavior of triple-negative breast cancer, a particularly therapy-resistant subclass of breast cancer [25]. Therefore, the development of therapies eliminating CD44(+)/CD24(-/low) CSCs or impeding activation of the signaling pathways these cells rely on may represent a promising approach for basal-like breast cancer.

CSCs reside in special niches, consisting of a specific cellular or extracellular matrix environment which determines the behaviour of the CSC via activation of specific signal transduction pathways [26]. Specifically, breast cancer stem cells are modulated by signal transduction pathways including the Wnt and IL-6/JAK2/STAT3 pathway [27,28]. Moreover, a positive correlation exists between the expression levels of IL-1alpha, IL-6, IL-8 and the CD44(+)/CD24(-/low) population in breast cancer cell lines [29]. A candidate molecule potentially modulating all of these pathways in breast cancer is the heparan sulfate proteoglycan Syndecan-1 (CD138). Syndecan-1 is predominantly expressed on epithelial cells and modulates numerous biological processes relevant to tumor progression [30]. It is a classical coreceptor for growth factors, angiogenic factors, morphogens and chemokines [31-33]. High expression of Syndecan-1 in breast cancer is associated with negative progostic parameters [34] and reduced breast cancer-specific overall survival [35]. Both Syndecan-1 and Wnt modulate the growth and differentiation of the mammary progenitor population [36]. Accordingly, resistance of Syndecan-1-deficient mice to experimentally induced tumorigenesis has been linked to an alteration of Wnt-responsive precursor cell pools [37,38], demonstrating the essential role of Syndecan-1 cancer stem cell function in a mouse model. 

Based on these findings, we hypothesize that Syndecan-1 may regulate human breast cancer stem cell function. To test this hyphothesis, we employ siRNA technology in the triple-negative MDA-MB-231 and in the hormone-receptor positive MCF-7 breast cancer cell lines to assess the molecular function of Syndecan-1. The modulatory role of Syndecan-1 on breast CSC properties is studied using flow cytometric analysis of SP, ALDH1 activity and the cell surface markers CD44 and CD24. Furthermore, we analyse the functional impact of Syndecan-1 depletion on the expression of effectors of the stemness-related IL-6/JAK2/STAT3 and Wnt signaling pathways, its developmental plasticity based on epithelial and mesenchymal marker expression and cell morphology, and its influence on the sphere and cyst formation capability in suspension culture. Our data demonstrate a role for Syndecan-1 in human breast CSC function, and underscore the importance of flow cytometry technology for the analysis of CSCs.

## Materials and Methods

### Antibodies and reagents

The antibodies p-STAT3 (Ser727, 6E4), STAT3 (79D7), p-NFκB p65 (Ser596, 39H1), and LRP-6 (C5C7) were from Cell Signaling (Beverly, MA). sIL-6R antibody and IL-6 were purchased from R&D Systems (Wiesbaden, Germany), Vimentin and GAPDH antibodies were from Santa Cruz Biotechnology (Santa Cruz, USA), and E-cadherin and tubulin antibodies from Sigma (Deisenhofen, Germany). CD44 and CD24 antibodies were obtained from Immunotools (Friesoythe, Germany). Media, fetal calf serum (FCS) and tissue culture supplies were from Gibco BRL (Karlsruhe, Germany). Unless stated otherwise, all chemicals were from Sigma.

### Cell culture

The human breast cancer cell lines MDA-MB-231 and MCF-7 were purchased from ATCC/LGC Promochem (Wesel, Germany). Cells were maintained in DMEM containing 10% FCS, 1% glutamine and 1% penicillin/streptomycin in a humidified atmosphere of 7% CO_2_ at 37°C and were cultured to 80% confluence and splitted two times a week. Sphere suspension cultures of MCF7 cells were performed in serum-free medium (DMEM, High Glucose, GlutaMAX™Gibco®), supplemented with B27 (Gibco®), 20 ng/mL EGF (Sigma) and 20 ng/mL bFGF (Immunotools), and 4 μg/mL heparin (Sigma) at a density of 10^3^ cells/mL. Cyst cultures of MCF7 cells were generated by placing single cells obtained from dissociated spheres into matrigel containing DMEM, High Glucose, GlutaMAX™Gibco®, 10% FBS supplemented with 0.1 g/ml human insulin (Sigma-Aldrich).

### Enrichment of MCF-7 cells with sphere formation capacity

A sub-population of MCF-7 cells with sphere formation capacity was enriched by generating spheres in a cell limiting dilution suspension condition which promotes the generation of spheres from single cells. Cultures were pipetted several times to allow spheres to be filtered from small cell aggregates with a 100 µm cell strainer. Single cells from enzymatically (0.25% Trypsin-EDTA) dissociated spheres were used to perform Syndecan-1 expression knock down with siRNA or transfection with a negative control siRNA. The average cell numbers for spheres of 4 and 7 days were between 8-32 and 64-256, respectively.

### siRNA knockdown of Syndecan-1 expression

siRNA knockdown was performed using siRNA #12634 (Ambion, Cambridgeshire, UK) targeting the coding region of Syndecan-1, and a negative control siRNA (negative control #1, Ambion). MDA-MB-231 and MCF-7 cells were transfected with 40nM siRNA using Dharmafect reagent (Dharmacon, Lafayette, CO, USA) according to the manufacturer’s instructions. Target downregulation was confirmed by flow cytometry. For suspension culture experiments, MCF7 cells were plated as adherent cells at a density of 1.1 × 10^5^ cells per 24 multiwell dish 1 day prior to transfection. The siRNA was transfected into the cells using the INTERFERin® reagent (Polyplus Transfection TM) according to the manufacturer’s instructions. The control and Syndecan-1 siRNA’s were used at a concentration of 25 nM unless indicated otherwise.  Cells were washed and trypsinized (0.25% Trypsin-EDTA) 72h after transfection, and re-plated as a suspension culture in low adhesion plates with a density of 10^3^ cells/mL to allow for sphere formation. The sphere forming efficiency is calculated as the number of spheres formed at day 7 relative to the initial number of cells plated in suspension (3.000).

### Flow cytometry

Flow cytometric confirmation of Syndecan-1 siRNA knockdown was performed essentially as previously described (39). Briefly, MDA-MB-231 breast cells treated with control and Syndecan-1 siRNA were harvested 72h after transfection by incubation with 2 mM EDTA in Ca^2+^/Mg^2+^-free PBS buffer for 10 minutes at 37°C with gentle agitation. 1x10^5^ cells in 100μl PBS/0.1% BSA (PBS/BSA) were incubated with 3μl anti-CD138-PC5 (clone BB4, Beckman Coulter, Krefeld, Germany) or mouse IgG1-PC5 control for 30 min at room temperature. Subsequently, cells were centrifuged (450g, 30 sec) and washed 2X with 300μl PBS/BSA. The cells were resuspended in 1 ml sheath fluid before analysis in a Beckman Coulter FC500 flow cytometer. Excitation took place with a 20 mW 488 nm laser and fluorescence emission was measured using a 675 nm bandpass filter. For the detection of stem cell specific cell surface markers, cells were incubated with 10µl of anti-CD44-APC, anti-CD24-PE and the APC and PE isotype controls for 30min at room temperature in the dark. Flow cytometric analysis took place on a CyFlow Space (Partec, Münster, Germany) equipped with a 25 mW 638 nm red laser diode and a 20 mW 488 nm blue laser, fluorescence emission was detected at 660 nm (BP675/20 nm) and 575 nm (BP 527/30 nm). Isotype controls were set in the first quadrant and gates were administered. CD44^-^ CD24^-^ resides in the Q3, CD44^+^ CD24^-^ in Q4, CD44^+^ CD24^+^ in Q2 and CD44^-^ CD24^+^ in Q1. CD24 expression was also measured over the whole population by setting a region-gate (RN1) in FL2. 

### Identification of ALDH-1 positive cells

ALDH-1 activity was assessed 72 hr after siRNA transfection by using the ALDEFLUOR^TM^ kit (StemCell Technologies, Köln, Germany) as previously described [40]. Briefly, 1x 10^6^ cells were resuspended in assay buffer containing ALDH substrate (1 µmol/L). Half of this suspension was used as a negative control and transferred into another tube containing 50 mmol/L of the specific ALDH-1 inhibitor diethylaminobenzaldehyde (DEAB). The cells were incubated for 1 hr at 37°C in a water bath in the dark and agitated every 10 minutes. After a final centrifugation at 400g for 5 min the cells were resuspended in 1 ml assay buffer and stored on ice prior to flow cytometry on a CyFlow Space (Partec) using the 488 nm blue laser for excitation. Fluorescence emission was measured at 545 nm (BP 527/30 nm). Gates were set by comparing the fluorescence of the DEAB control with that of the original sample. 

### Side population analysis

Side population (SP) analysis was performed 72 hr after control and Syndecan-1 siRNA transfection using the Hoechst 33342 dye exclusion technique as previously described [40]. 1x10^6^ cells were incubated in DMEM containing 2% (v/v) FCS for 90 min at 37°C either with 5 µg/mL Hoechst 33342 (Sigma-Aldrich) or in the presence of 50 µM verapamil (Sigma-Aldrich). Finally, 2 µg/mL propidium iodide was added for cell death discrimination, and cells were stored on ice until analysis. Cells were analysed on a CyFlow Space (Partec) using a 16 mW 375 nm UV laser for excitation, emission was measured at 475 nm (BP 455/50) and at 665 nm (LP 665 nm). Signals were slivered by a dichroic mirror of 610 nm to measure Hoechst signal intensity in both channels. All cells with a low Hoechst fluorescence and which were not visible in the verapamil control were gated (R2) as SP cells. SP analysis was further combined with the CD44/CD24 analysis. In this case the SP staining was done first and followed by the cell surface marker protocol. SP cells and non-SP cells were sorted and mRNA was isolated to determine the expression of Sdc-1 in both populations.

### Western blot

30-50μg of protein/lane was separated on 10% gels and electrotransferred to Hybond nitrocellulose membranes (Amersham). Antibody incubations were performed essentially as described [32]: Following 1h blocking, detection of phosphorylated STAT3 was performed using mouse primary antibodies (1:1000) and HRP–conjugated anti-mouse IgG antibodies (Calbiochem, 1:10000). Subsequently, membranes were subjected to an ECL reaction and signal quantification with NIH ImageJ software, normalizing the densitometric values of p-STAT3 to tubulin (as loading control). For tubulin and GAPDH detection, membranes were stripped with 0.2 M glycin buffer (pH 2.5), washed, and reincubated with primary antibody followed by the procedure described above. p-NFkB, STAT3, LRP-6, sIL-6R, E-cadherin, and vimentin were detected analogously using the appropriate primary and HRP-coupled secondary antibody pairs.

### RT-PCR analysis

Total cellular RNA was isolated using rna-OLS (OMNI Life Science, Hamburg, Germany) and reverse transcribed (Advantage First strand cDNA synthesis kit; Fermentas Life Science, Leon-Rot, Germany) and amplified in a BioMetra PCR reactor (BioMetra, Göttingen, Germany). A DNA fragment corresponding to base pairs 1233–1303 of human IL-6 mRNA was amplified using the primers 5'-GAGAAAGGAGACATGTAACAAGAGT-3' and 5'- GCGCAGAATGAGATGAGTTGT- 3' (170 bp) and the following amplification cycle: 94°C for 4 min; 27 cycles of 94°C for 30 s, 60°C for 30 s and 72°C for 30 s; and 72°C for 5 min. The primers 5'-ATGCTGGCCGTCGGCTGCGCGCTG-3' and 5'- TCTGAGCTCAAACCGTAGTCT- 3' were used to amplify a PCR fragment corresponding to base pairs of human IL-6R (768bp). Samples were amplified for 32 cycles of 1 min at 95°C followed by 90 s annealing at 55°C followed by 90 s of extension at 72°C. Upon completing the final cycle, samples were incubated for 10 min at 72°C. The primers for human CCL20 (390bp) 5'-ACCATGTGCTGTACCAAGAGTTTG-3' and 5'-CTAAACCCTCCATGATGTGCAAGTGA-3'. The amplification profile was 30 cycles of 45-s denaturation at 95°C and 2.5-min annealing and extension at 60°C for cell lines and tissues. The housekeeping gene β-actin was used as an internal control and amplified using the primers 5'-CAAAGACCTGTACGCCAACAC-3' and 5'-CATACTCCTGCTTGCTGATCC-3', corresponding to base pairs 943–1220 of human β-actin and the following amplification cycle: 95°C for 3 min; 18 cycles of 95°C for 1 min, 56°C for 1 min and 72°C for 1 min; and 72°C for 10 min. PCR products were subjected to gel electrophoresis on 1.5% agarose gels. After ethidium bromide staining, DNA bands were photographed under UV illumination using a BioDoc Analyze system (Biometra). Scanned bands were analyzed using the Image J software (NIH, Bethesda, MA, USA) and signal intensities were normalized for actin expression. 

### Confocal immunofluorescence microscopy

50,000 cells were grown on coverslips for 16 hr in six-well plates, fixed with 3.7% PBS-buffered formaldehyde, and permeabilized with PBS/0.1% Triton-X100. Nonspecific binding was blocked with PBS/1% Aurion BSA-c (DAKO, Glostrup, Denmark). Coverslips were subsequently incubated for 1 hr with ALEXAFluor-568-labeled phalloidin (Invitrogen, 1:1,000). Slides were analyzed with an LSM-510-META confocal microscope (Carl Zeiss, Jena, Germany) equipped with a Plan10 Apochromat 63x/1.4 oil immersion objective.

### Statistical analysis

Unless indicated otherwise, data were analysed using the unpaired two-tailed Student’s t-test. A P-value <0.05 was considered statistically significant.

## Results

### siRNA-mediated silencing of Syndecan-1 reduces the side population and ALDH1 activity in MDA-MB-231 and MCF-7 breast cancer cells

**Figure 1:**
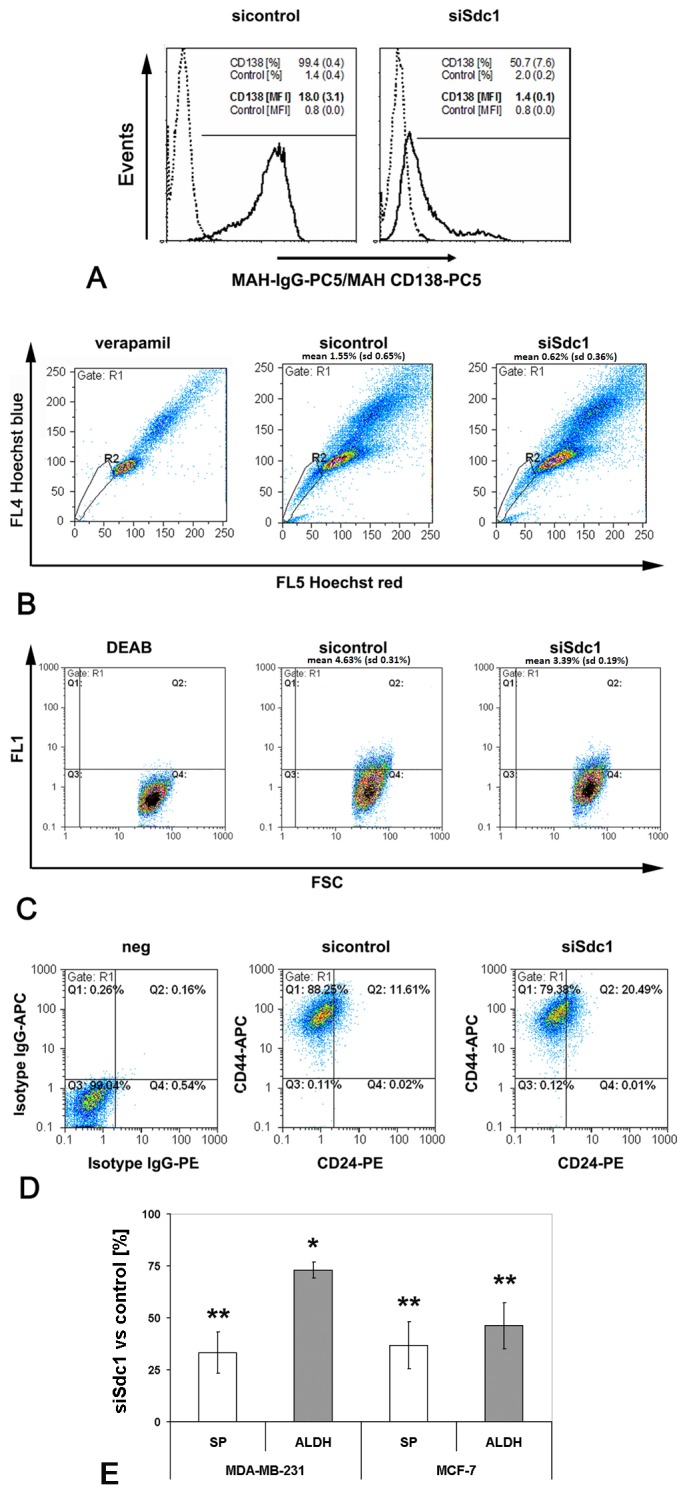
Flow cytometric analysis of putative cancer stem cell pools in control and Syndecan-1-silenced MDA-MB-231 breast cancer cells. Syndecan-1 silencing reduces the SP phenotype, ALDH1 activity, and the CD44(+)/CD24(-/low) phenotype. A) Confirmation of Syndecan-1 siRNA knockdown by flow cytometry. The percentage of marker-positive cells and the mean fluorescent intensity of events (mean (SD), n=4) are depicted on the plots. (Dotted lines: mouse-IgG1-PC5 control; solid lines: anti-CD138-PC5; marker for percentage of CD138-positive cells; MFI = median fluorescent intensity). B) Side population analysis. Control and Syndecan-1-depleted cells were incubated with the Hoechst 33342 dye in the presence or absence of 50 µM verapamil, and subjected to flow cytometry. Gate R1 shows SP cells. C) Example of the identification of putative cancer stem cells displaying aldehyde dehydrogenase (ALDH) activity. Control and Syndecan-1 knockdown cells were incubated with fluorescent ALDH substrate in the presence or absence of the inhibitor diethylaminobenzaldehyde (DEAB), followed by flow cytometric analysis. D) Example of flow cytometric analysis for the cell surface expression of CD44 and CD24 protein. Control and Syndecan-1 knockdown cells were incubated with isotype IgG-PE and-APC, CD24-PE and CD44-APC antibodies followed by sorting the cells by flow cytometry into left lower quadrant CD44(-)CD24(-),left upper quadrant CD44(+)CD24(-), right lower quadrant CD44(-)CD24(+), right upper quadrant CD44(+)CD24(+). A-D) Representative examples of n≥3 experiments. E) Quantitative analysis of SP and ALDH measurements in Syndecan-1 and control siRNA treated MDA-MB-231 and MCF-7 cells. Syndecan-1 siRNA knockdown results in a significant decrease of the SP and ALDH activity. Data are expressed as mean percentage +/- SEM relative to the controls (set to 100%). (n=3-5, *=P<0.05, **=P<0.01).

To investigate the impact of Syndecan-1 on CSC properties, we used a transient transfection siRNA approach. Successful silencing of Syndecan-1 in Syndecan-1 siRNA vs control siRNA-transfected MDA-MB-231 cells was confirmed by flow cytometry (Figure 1A). We next investigated the SP phenotype, a surrogate marker for putative stem cell activity based on the ability of effluxing the fluorescent dye Hoechst 33342 via ABC transporters [20,21], in the Syndecan-1-depleted cells. Hoechst 33342 dye exclusion assays revealed that Syndecan-1 siRNA transfected cells contained on average 0.62% (±0.36%) SP cells, whereas control cells contained 1.55% (±0.65%) SP cells (Figure 1B), corresponding to a significant >60% relative decrease of the SP upon Syndecan-1 depletion (p<0.01, n=3) (Figure 1E). These data demonstrate a considerable impact of Syndecan-1 depletion on the CSC SP. Side population cells were sorted and Sdc-1 expression was determined by qPCR on mRNA level and by flow cytometry on protein level in comparison to the non-SP population. The fold change of gene expression was 1.3 for the SP+ population and thus, slightly increased while the protein level was the same in both sorted populations (data not shown). We next analyzed an additional breast cancer stem cell-associated parameter [23,41], the activity of ALDH isoform 1 (ALDH1). Flow cytometry-based ALDH1 activity analysis revealed that Syndecan-1-silenced cells had a pool of 3.39% (±0.19%) ALDH1-positive cells compared to a pool of 4.63% (±0.31%) ALDH1-positive cells in controls, corresponding to a significant relative decrease by >25% upon Syndecan-1 depletion (P<0.01, n=4) (Figure 1E). When an additional breast cancer cell line, MCF-7, was analyzed for its cancer stem cell pool, Syndecan-1 depleted cells revealed 0.31% (±0.3%) SP cells compared to 0.68% (±0.5%) in the control-siRNA transfected cells, corresponding to a Syndecan-1-dependent relative decrease by >40% (P<0.01, n=4) (Figure 1E). Also, the ALDH1 positive fraction was lower in the Syndecan-1-silenced cells (0.69% (±0.6%)) compared to the control-siRNA treated cells (1.44% (±0.9%)), resulting in a significant relative decrease by >50% (P<0.05, n=5) (Figure 1E, Table 1). 

**Table 1:** Overview on proportion of cells with different stem cell properties for MCF-7 and MDA-MB-231 cells.

**Cell line**	**Control siRNA**			**Sdc-1 siRNA**		
	SP	ALDH	CD44+/CD24-	SP	ALDH	CD44+/CD24-
MDA-MB-231	1.55% (±0.65%)	4.63% (±0.31%)	85.60% (±2.13%)	0.62% (±0.36%)	3.39% (±0.19%)	79.67% (±2.81%)
MCF-7	0.68% (±0.5%)	1.44% (±0.9%)	0,56% (±0,71%)	0.31% (±0.3%)	0.69% (±0.6%)	1,11% (±1,43%)

Data are shown as mean ± SD from at least 3 independent experiments.

### siRNA-mediated Syndecan-1-depletion reduces the CD44(+) CD24(-) pool and increases the CD44(+) CD24(+) population in MDA-MB-231 cells

**Figure 2:**
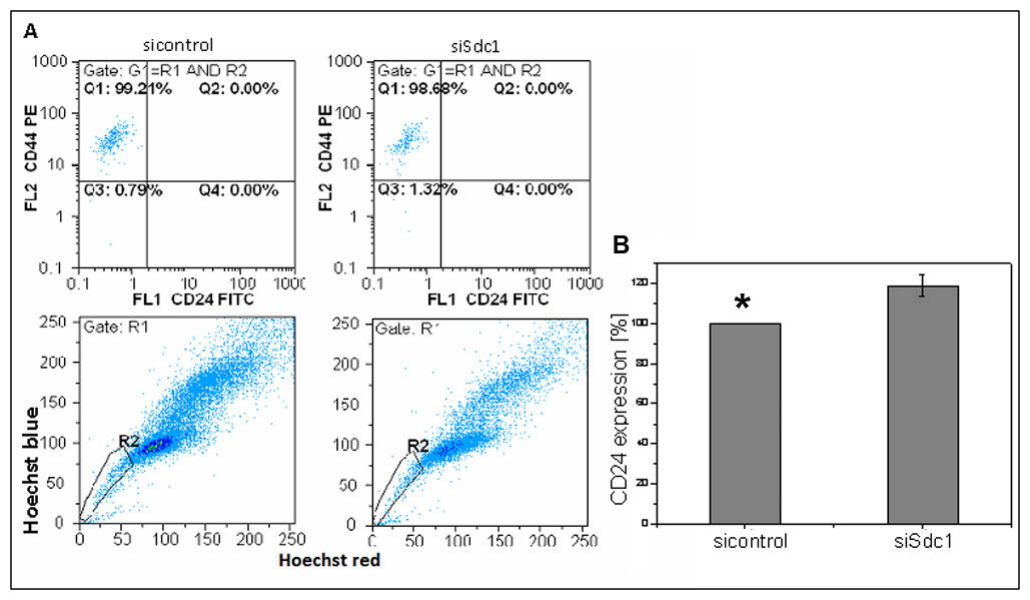
Characterization of SP cells concerning their CD44/CD24 expression. The combination of SP- and CD44/CD24 measurement revealed a proportion of 99.2% of SP cells with the CD44+/CD24(-/low) phenotype after control siRNA treatment while the Syndecan-1 knockdown approach showed that this proportion decreased only slightly to 98.7% (A). Influence of Syndecan-1 knockdown on CD24 expression. Compared to the control-siRNA treated cells, the Syndecan-1-siRNA transfected cells showed a 20% (±8.0%) increase in CD24 expression (*=P<0.05, n=3) (B).

Breast cancer stem cells can be identified by a CD44(+) CD24(-/low) phenotype that augments mammosphere formation in vitro and exhibit increased resistance to chemo- and radiotherapy [24,25,42]. To explore the impact of Syndecan-1 silencing on this phenotype, expression of CD44 and CD24 was analyzed by flow cytometry in control and Syndecan-1-siRNA transfected MDA-MB-231 cells (Figure 1D). Syndecan-1 silencing reduced the CD44(+) CD24(-) phenotype from 85.60% (±2.13%) in control cells to 79.67% (±2.81%) in Syndecan-1-depleted cells (P<0.05, n=4). The number of CD44(+) CD24(+) cells increased non-significantly from 10.61% (±3.48%) in controls to 18.86% (±2.97%) in Syndecan-1-silenced cells. However, increase of CD24 expression through Syndecan-1 silencing was also noted when measured over the whole population by calculating the mean x-value in FL2. Compared to the control-siRNA treated cells, the Syndecan-1-siRNA transfected cells showed a significant 20% (±8.0%) increase in CD24 expression (n=3, P<0.05) (Figure 2B). Furthermore, our analysis unveiled a subset of 3.70% (±1.98%) CD44(-) CD24(-) cells in controls, compared to 1.33% (±0.50%) in the Syndecan-1-silenced cell line (n.s.). A minor fraction of 0.13% (±0.11%) CD44(-) CD24(+) cells was found in controls, that did not substantially differ from Syndecan-1-depleted cell pools (0.10% ±0.08%) (n.s.). By combining SP and surface marker expression analysis, about 99.5% of the SP positive cells revealed the CD44(+)/CD24(-) phenotype in the control siRNA treated cells compared to 98.7% in the Syndecan-1 siRNA treated cells (Figure 2A). Compared to MDA-MB-231 cells, MCF-7 cells contained only a minor pool of about 1% CD44(+) CD24(-) cells, which was not significantly altered by Syndecan-1 siRNA treatment (Table 1).

### Syndecan-1 silencing impairs the formation of spheres and differentiation into cysts in MCF-7 cells

**Figure 3:**
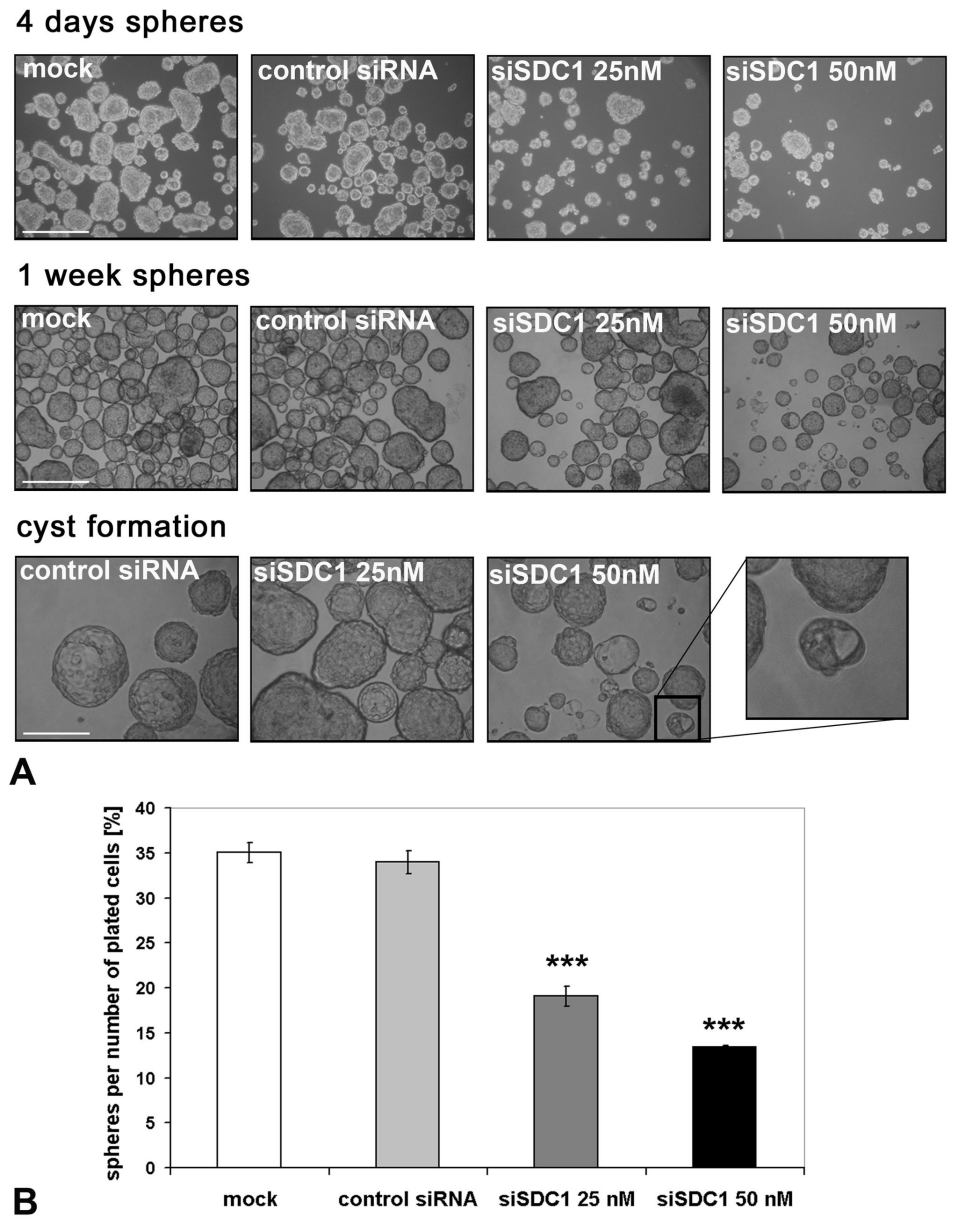
Syndecan-1 silencing impairs the formation of spheres and differentiation into cysts in MCF-7 cells. A) MCF-7 with sphere formation capacity were enriched for and transfected with a control siRNA or Syndecan-1 siRNA, followed by placing the cells in non-adherent culture conditions that promote sphere formation from single cells. After 4 days in suspension without siRNA it’s evident that Syndecan-1 knockdown is affecting proliferation. At this step it is already visible that the spheres are not only smaller but also more irregular compared to controls (upper panel). After 1 week, the spheres formed by Syndecan-1 transfected MCF-7 are less abundant, smaller and there are much more aggregates compared to controls (central panel). After 1 week in suspension culture, MCF-7 cells start to form cysts (bottom panel). The Syndecan-1-transfected cells show a drastically reduced cyst formation capability. The insert shows a Syndecan-1-depleted sphere that is generating a small “sphere/cyst hybrid”. Scale bar = 500µm (upper, central panels); 200µm (lower panel). B) Quantitative analysis of the sphere formation efficiency in control and Syndecan-1 siRNA-treated MCF-7 cells. Sphere number was determined 1 week after plating 3.000 transfected cells. Syndecan-1 siRNA-treatment results in a significant reduction of sphere formation efficiency (P<0.001, n=6).

Several studies have demonstrated that sphere formation is a property associated with stem cells of various tissues, including the mammary gland [5,43,44]. Our flow cytometric analysis indicated that siRNA-mediated depletion of Syndecan-1 may result in a loss of stem cell properties. To address this question, we enriched for MCF-7 with sphere formation capacity and transfected the cells with a control siRNA or Syndecan-1 siRNA, followed by placing the cells in non-adherent culture conditions that promote sphere formation from single cells. It was observed that 72 hours after transfection, while proliferation was not affected in mock-transfected cells, and control siRNA transfection displayed an 8% inhibition of proliferation, Syndecan-1 siRNA transfected induced an inhibition of 78% (data not shown). After 4 days in suspension culture, sphere formation was visible in the mock and control siRNA-transfected cells. The few spheres that were observed with Syndecan-1-depleted cells were not only smaller but also irregular in shape, suggesting that sphere formation process was severely affected (Figure 3A). After 1 week in suspension culture, Syndecan-1-depleted sphere numbers were showed a tendency to aggregate, suggesting that the cells within the spheres had lost the capacity to sustain their morphology (Figure 3A). While mock and control siRNA-treated cells started to differentiate into cyst structures that are representative of alveoli, this process was not observed with MCF-7 cells transfected with Syndecan-1 siRNA (Figure 3A). A quantitative analysis of the sphere formation efficiency revealed a significant reduction by >50% upon Syndecan-1 siRNA knockdown (Figure 3B).

### Syndecan-1 silencing induces downregulation of components of the IL-6 signaling pathway in MDA-MB-231 cells

**Figure 4:**
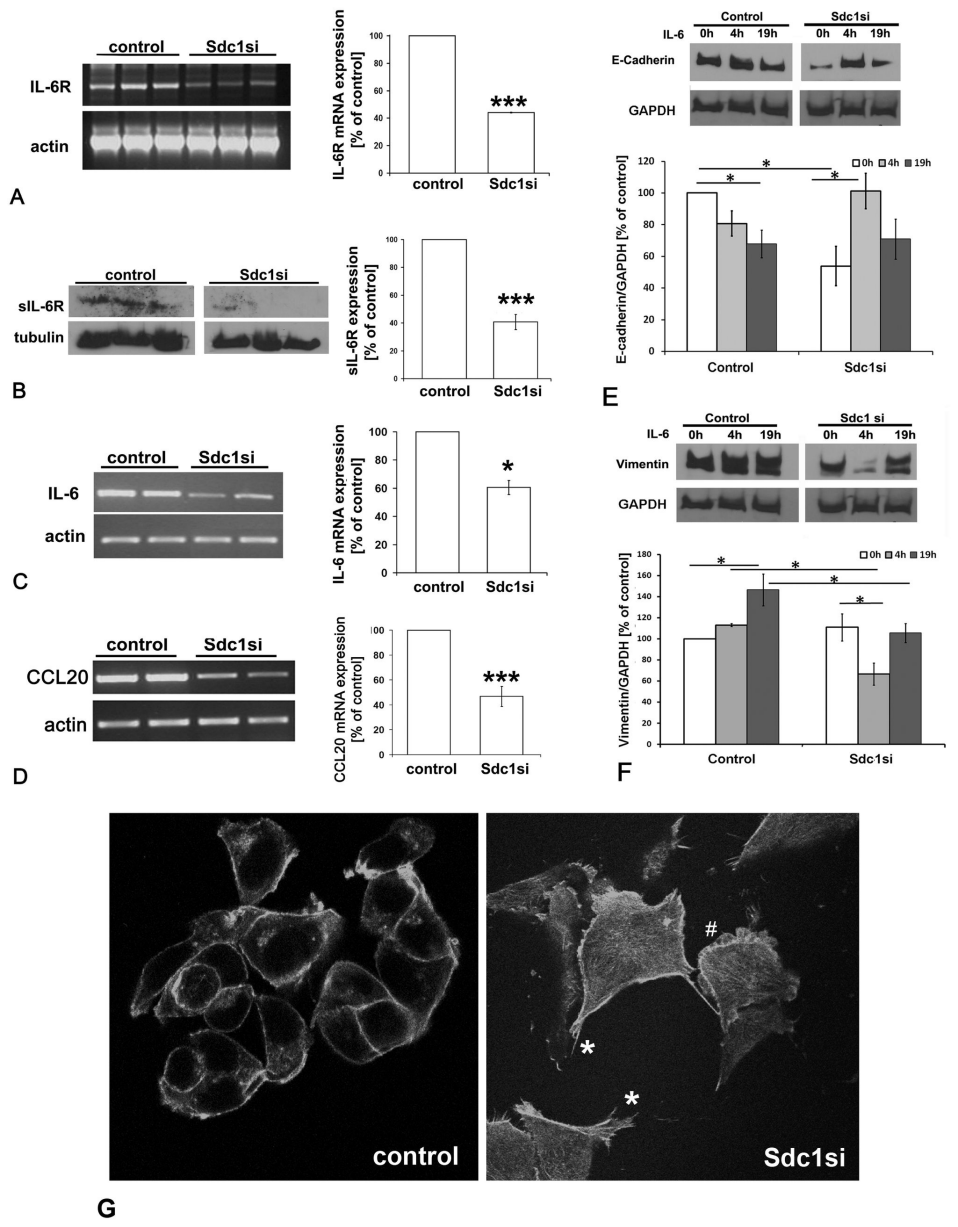
siRNA-mediated knockdown of Syndecan-1 downregulates expression of IL-6R, IL-6 and CCL20 and dysregulates epithelial and mesenchymal marker protein expression in MDA-MB-231 breast cancer cells. A) left panel: RT-PCR analysis of IL-6R expression in MDA-MB-231 cells subjected to Syndecan-1 siRNA knockdown. Following total RNA isolation, mRNA was reverse transcribed and used as a template for PCR amplification of IL-6R. Right panel: PCR band intensities were normalized for actin expression and the data were analyzed using the paired Student's *t*-test. B) Left panel: Western blot analysis reveals reduction of IL-6R following Syndecan-1 silencing. Lysates of control and Syndecan-1 silenced cells were collected and 30-50µg protein/lane was immunoblotted and probed with sIL-6R antibody. Right panel: Immunoblot band intensities were normalized for tubulin expression and the data were analyzed using the paired Student's *t*-test. Data shown are triplicates from a single experiment representative of three independent experiments. C) left panel: RT-PCR analysis of IL-6 expression. Right panel: semiquantitative densitometric analysis (see panel A). D) left panel: RT-PCR analysis of CCL20 expression. Right panel: semiquantitative densitometric analysis (see panel A). *=p<0.05, ***=p<0.001, n≥3, error bars=SEM. E,F) The influence of IL-6 treatment on the expression of the epithelial marker E-cadherin (E) and the mesenchymal marker vimentin (F) was studied by Western blotting. Cells were stimulated by 50ng/ml IL-6 24h after transfection with siRNA for 4h and 19h. In control cells, IL-6 treatment for 19h promoted EMT. Syndecan-1 depletion resulted in significant downregulation of E-cadherin expression. IL-6 treatment of Syndecan-1 depleted cells for 4h resulted in marker expression changes suggestive of enhanced mesenchymal-to-epithelial transition. (E,F) Upper panels = representative Western blots, lower panels = quantitative analysis. n≥3,*=P<0.05. G) Confocal immunofluorescence microscopy of phalloidin-labeled actin filaments reveals increased formation of actin stress fibers, filopodia (*) and lamellopodia (#) in Syndecan-1 siRNA-treated compared to control siRNA treated MCF-7 cells.

Among the signal transduction pathways modulating CSC properties, the IL-6/JAK/STAT3-pathway is active in CD44(+) CD24(-) breast CSCs [28]. As we had previously observed a Syndecan-1-dependent dysregulation of IL-6 expression in experimental models of inflammation and decidualization [45-47], we next investigated whether Syndecan-1-depletion affects expression of IL-6 and its receptor IL-6R using PCR and Western blot analysis, respectively. Compared to controls, IL-6 mRNA expression was significantly suppressed by 40% in MDA-MB-231 Syndecan-1 siRNA transfectants, whereas IL-6R expression was significantly reduced 56% and 60% at the mRNA and protein levels, respectively (Figure 4A,B). Since IL-6 expression can be induced by the chemokine CCL20 (MIP3α) [48], a modulator of mesenchymal stem cell differentiation [49], we investigated its expression in Syndecan-1-silenced and control cells at the transcriptional level. RT-PCR analysis revealed a substantial downregulation of CCL20 mRNA by 53% upon Syndecan-1-depletion (Figure 4D). In contrast to MDA-MB-231 cells, IL-6R, IL-6 and CCL20 expression were not downregulated upon Syndecan-1 knockdown in MCF-7 cells (results not shown). As IL-6 is known to modulate developmental processes including epithelial-to-mesenchymal-transition (EMT) in a variety of experimental systems [50,51], we studied the influence of Syndecan-1 depletion and IL-6 on this process in vitro. EMT is an important event in the progression of breast cancer [51,52]. E-cadherin and vimentin have been frequently used as markers for the epithelial and mesenchymal lineage, respectively, in this context. While loss of E-cadherin causes dysfunction of the cell-cell junction system, thus triggering cancer cell invasion and metastasis, vimentin is a ubiquitous mesenchymal intermediate filament supporting mechano-structural integrity of quiescent cells [50-52]. Previous data indicated a significant downregulation of E-cadherin upon Syndecan-1 siRNA knockdown in MDA-MB-231 and MCF-7 cells [39], but the impact of IL-6 on this process is unclear. MDA-MB-231 cells were subjected to control and Syndecan-1 siRNA treatment and incubation with IL-6 for 0h, 4h and 19h, respectively. Western blot analysis of control cells demonstrated that IL-6 treatment for 19h resulted in a significant downregulation of E-cadherin, and a significant upregulation of vimentin expression, suggesting that IL-6 promotes EMT in control cells (Figure 4E,F). Syndecan-1 siRNA knockdown resulted in substantially reduced E-cadherin levels (Figure 4E) [39], whereas vimentin expression was not affected. IL-6 treatment of Syndecan-1 depleted cells for 4h resulted in a substantial and significant upregulation of E-cadherin and a corresponding downregulation of vimentin expression, suggesting that IL-6 may reverse EMT in the absence of Syndecan-1 in MDA-MB-231 cells (Figure 4E,F). In accordance with previous reports [53] vimentin was not expressed in MCF-7 cells, and no induction of vimentin expression was observed upon Syndecan-1 siRNA knockdown (data not shown). qPCR investigation of additional markers of EMT, ZEB2 and SNAI1, revealed only fluctuations, but no significant expression differences between Syndecan-1 knockdown and control MDA-MB-231 and MCF-7 cells (Figure S1). Regarding the acquisition of a migratory phenotype, we had previously demonstrated increased filopodia formation in Syndecan-1-depleted MDA-MB-231 cells [39]. In accordance with these findings, confocal immunofluorescence microscopy investigation of cell morphology by phalloidin staining of the actin cytoskeleton revealed increased formation of actin stress fibers, filopodia and lamellopodia in MCF-7 cells subjected to Syndecan-1 siRNA treatment relative to controls (Figure 4G). 

### Constitutive activation of STAT3 and NFkB signaling in MDA-MB-231 cells is reduced upon Syndecan-1 siRNA knockdown

**Figure 5:**
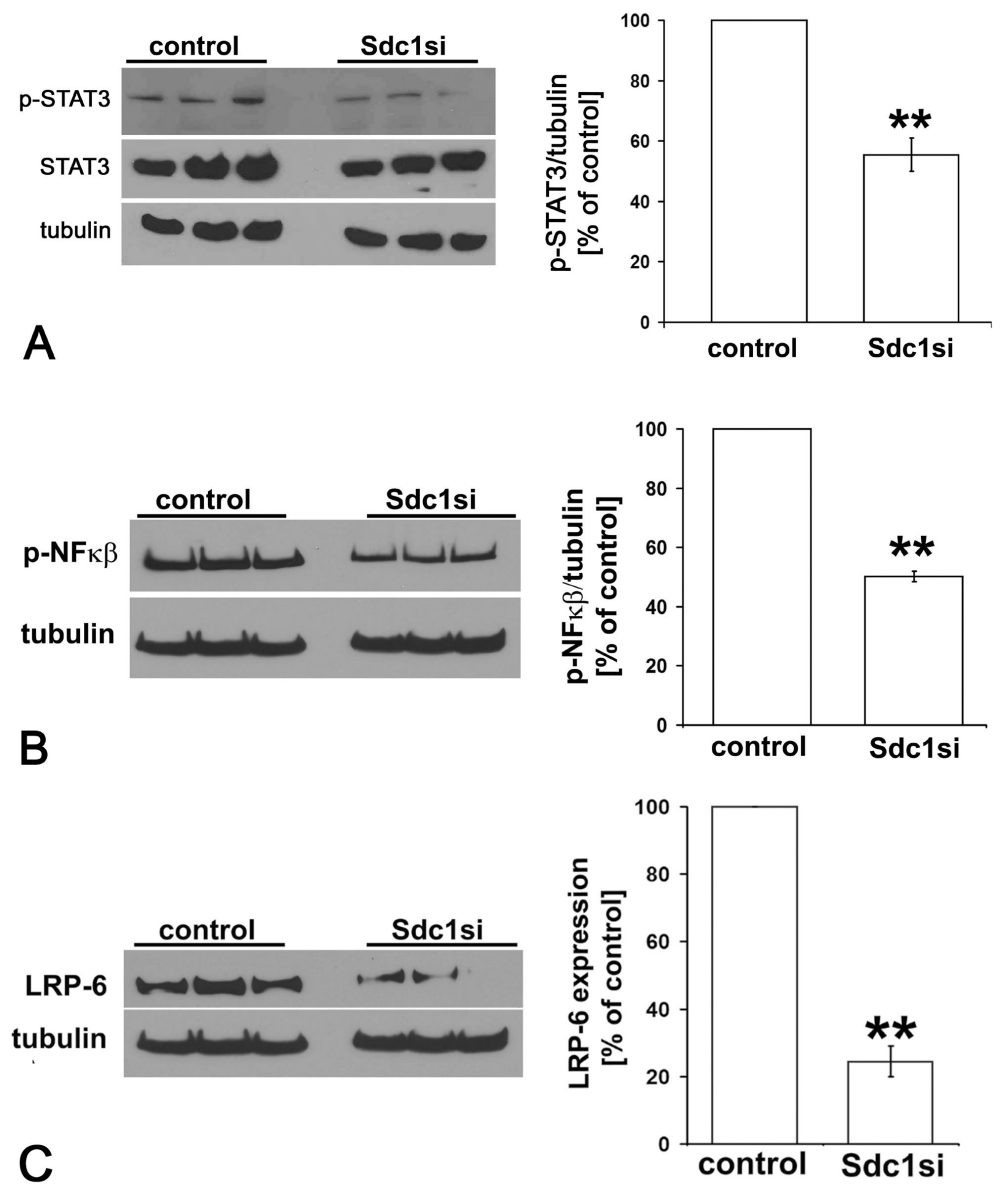
Syndecan−1 modulates activation of the STAT3 and NFκB signaling pathways and expression of LRP-6 in MDA−MB−231 breast cancer cells. Lysates of control and Syndecan−1 silenced cells were collected and 30-50µg protein/lane was immunoblotted and probed with the indicated antibodies. A) Western blot analysis reveals reduction of the phosphorylated form of STAT3 in Syndecan−1−silenced cells compared to controls. B) Syndecan−1 depletion leads to a significant reduced activation of NFκB. Immunoblot band intensities were normalized for tubulin expression and the data were analyzed using the paired Student's *t*-test. Data shown are triplicates from a single experiment representative of three independent experiments. **=p≤0.01, n≥3, error bars=SEM. C) Lysates of control and Syndecan−1 silenced cells were collected and 30-50µg protein/lane was immunoblotted and probed with an antibody recognizing LRP-6 (left panel). Immunoblot band intensities were normalized for tubulin expression and the data were analyzed using the paired Student's *t*-test (right panel). Data shown are triplicates from a single experiment representative of three independent experiments. **=p≤0.01, n≥3, error bars=SEM.

IL-6 / IL-6R-mediated signal transduction involves activation of the transcription factor STAT3, a pathway upregulated in putative breast CSCs capable of mammosphere formation [54]. In addition, NFκB, an antiapoptotic transcription factor regulating IL-6 expression, is constitutively activated in breast cancer and contributes to CSC stemness and chemoresistance [55]. We analyzed the functional impact of siRNA-mediated Syndecan-1 knockdown on the activation status of STAT3 and NFκB by Western blotting using phosphorylation-specific antibodies. Syndecan-1-silencing lowered the cellular amounts of phosphorylated STAT3 by 45% and of activated NFκB by 50% (Figure 5A,B). Taken together, these findings suggest that Syndecan-1 plays a critical role in modulating the IL-6/STAT3 pathway, contributing to a reduction of the MDA-MB-231 CSC pool as shown by flow cytometric SP and ALDH1 analyses (Figure 1). 

### Syndecan-1 knockdown is associated with reduced expression of the Wnt-coreceptor LRP-6

The Wnt/β-catenin signaling pathway is associated with sustained breast CSC function [36,56]. Notably, expression of the Wnt coreceptor LRP-6 is frequently upregulated in the particularly aggressive tumor entity of triple negative breast cancer [55]. Since Syndecan-1-deficient mice were proposed to be resistant to mammary tumor formation due to reduced Wnt-1 responsive progenitor cell pools, we investigated the influence of Syndecan-1 siRNA knockdown on the expresssion of Wnt-1, its coreceptor LRP-6, and the target of canonical Wnt-signaling, ß-catenin, in the triple negative MDA-MB-231 cell line. Syndecan-1 silencing led to a significant and substantial downregulation of the Wnt-coreceptor LRP-6, as demonstrated by Western blotting (Figure 5C). Investigation of membrane fractions of Syndecan-1-depleted MDA-MB-231 and MCF-7 cells likewise revealed downregulation of LRP-6 (Figure S2). In contrast, qPCR revealed that Wnt-1 transcripts levels were not altered by Syndecan-1 knockdown (results not shown). Overall, these findings suggest that Syndecan-1 might modulate the progenitor cell response to Wnt-1 via regulation of LRP-6 expression. 

## Discussion

In the present study, we have combined flow cytometry analysis with a molecular biological approach to demonstrate a role for the heparan sulfate proteoglycan Syndecan-1 in human breast CSCs. Flow cytometry revealed that siRNA-mediated Syndecan-1 silencing in the triple negative MDA-MB-231 cell line resulted in a reduced SP and a reduced ALDH1-positive cell pool. The results were confirmed in hormone receptor positive MCF-7 cells. Furthermore, Syndecan-1 knockdown significantly reduced the CD44(+) CD24(-) and increased the CD44(+) CD24(+) phenotype in MDA-MB-231 cells. These findings mark Syndecan-1 as an important contributor to CSC stemness, and are in accordance with the observed upregulation of Syndecan-1 in breast cancer [34,35] and resistance of Syndecan-1-deficient mice to experimentally-induced breast cancer [36-38]: CSCs are thought to be enriched in the SP, which is responsible for increased therapy resistance [3,21]. Notably, a CD44+/CD24(-/low) gene expression signature is enriched in the SP [57]. About 99.5% of SP cells belong to the CD44+/CD24(-/low) fraction in the control-siRNA treated cells and this relation is not significantly changed by a Syndecan1 knockdown (98.7%). Therefore, the CD44+/CD24(-/low) phenotype appears to cosegregate with the reduced SP in the Syndecan-1 depleted MDA-MB-231 cells. In addition, ALDH1 activity is associated with a breast CSC phenotype and poor survival in breast cancer patients [41]. CD44(+) CD24(-) cells are associated with basal-like and mesenchymal cancers, and ALDH1 positive cells are more common in HER2-positive and basal-like tumors than in tumors of the luminal subtype, providing a link between the CSC markers affected by Syndecan-1 depletion [57-59]. Controversial reports on the association of a CD44(+) CD24(-/low) phenotype and poor prognosis of breast cancer have been reported. A recent study on 35 breast tumors of the luminal subtype showed that CD44(+) CD24(-/low) tumors were significantly associated with axillary lymph node metastasis compared with those of CD44(+) CD24(+) type [60]. These findings support our data and are in line with an *in vitro* study demonstrating that the CD44(+) CD24(-) subpopulation of MDA-MB-231 cells has a higher invasive capacity than the CD44(+) CD24(+) phenotype [29], and the observation of increased invasiveness of Syndecan-1-depleted MDA-MB-231 cells [39]. In contrast, a CD44(-) CD24 (+) phenotype is a poor prognostic marker in early invasive breast cancer [61]. Lack of CD44 expression is associated with lymph node involvement, regardless of CD24 status, whereas the lack of both CD44 and CD24 is connected with high histologic grade and unfavorable prognosis [62], suggesting that the role of the CD44(+) CD24(-/low) breast CSC population might be stage-dependent. 

Notably, the reduced expression of stem cell markers in Syndecan-1 depleted cells was associated with a functional loss of stem cell properties: siRNA knockdown in MCF-7 cells significantly reduced their capability to form spheres in nonadherent culture, and impaired differentiation into cysts. Moreover, Syndecan-1-depletion resulted in a dysregulation of the IL-6-induced shift in E-cadherin and vimentin expression in MDA-MB-231 cells, emphasizing the potential importance of Syndecan-1 for a biological process with relevance for physiological developmental processes and breast cancer metastasis alike [63]. Some of the observed changes are suggestive of a potential induction of EMT in Syndecan-1-depleted cells, such as the downregulation of E-cadherin, the acquisition of a migratory phenotype in MCF-7 and MDA-MB-231 [39] cells, and the upregulation of vimentin in MDA-MB-231 cells. This process would be conform with previous data in non-malignant cells and tissues, such as the loss of Syndecan-1 and E-cadherin during epithelial-mesenchymal transformation in the embryonic palate [64], or the induction of an EMT-like process in normal murine mammary gland epithelial cells upon Syndecan-1 antisense RNA treatment [65]. However, lack of significant changes in additional EMT marker expression (ZEB2, SNAI1), and a failure to induce vimentin upregulation in MCF-7 cells by Syndecan-1-depletion suggest that loss of Syndecan-1 is not fully associated with the classical process of EMT. As EMT-like changes were more prominent in mesenchymal-like MDA-MB-231 compared to more epithelial-like MCF-7 cells, we speculate that downregulation of Syndecan-1 may be important for driving the mesenchymal phenotype once it is established, but that it may not be sufficient to induce a full EMT.

**Figure 6:**
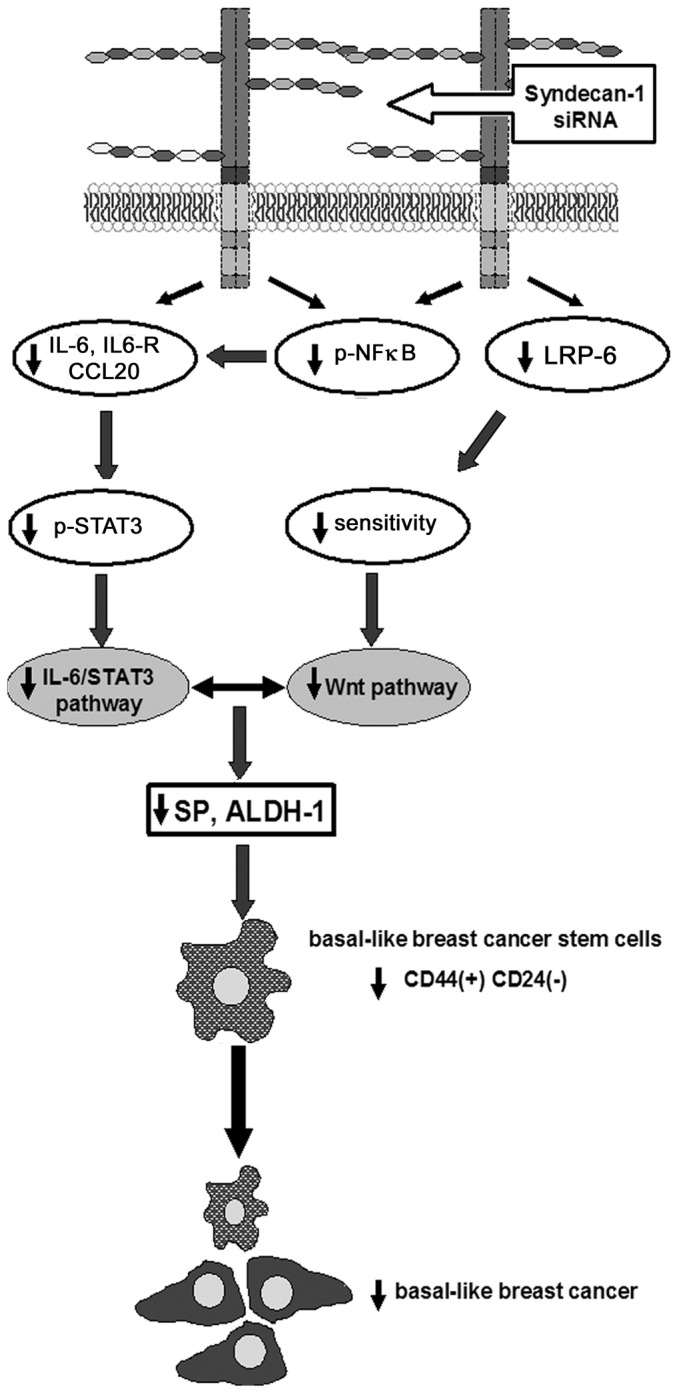
Syndecan-1 modulates triple-negative breast cancer stem cell properties via the IL-6/STAT3, NFkB and Wnt signaling pathways. siRNA-mediated knockdown of Syndecan-1 expression results in decreased expression of IL-6, the IL-6R and the chemokine CCL20, possibly due to reduced activation of NFkB. Reduced expression of components of the IL-6 signaling pathway results in decreased constitutive activation of STAT3 in MDA-MB-231 cells. Low expression of the Wnt-coreceptor LRP-6 in Syndecan-1-deficient cells may reduce responsiveness to Wnt signaling. The attenuation of mutiple stemness-related signaling events results in a reduction of the SP and ALDH-activity, two surrogate parameters of stem cell activity. The reduction of the CD44(+)/CD24(-/low) phenotype may have implications for novel Syndecan-1-centered therapeutic approaches of basal-like breast cancer.

To complement the flow cytometric characterization of the Syndecan-1-dependent CSC phenotype, we have analyzed signal transduction pathways potentially modulated by this heparan sulfate coreceptor [31,33]. Inflammatory signaling pathways have been linked to breast CSCs [66]. Of particular interest in this respect is the transcription factor NFkB, which regulates expression of a wide range of proinvasive and inflammatory cytokines including IL-6 and CCL20, thus acting as a potential target for the inhibition of breast CSCs [67-69]. Interestingly, the IL-6 / STAT-3 pathway is an essential signaling pathway that induces or maintains the CD44(+) CD24 (-/low) CSC phenotype [28,54,70]. According to our findings, Syndecan-1 silencing in triple-negative MDA-MB-231 breast cancer cells inhibited proinflammatory signaling via downregulation of relevant receptors and ligands (IL-6/IL6-R/CCL20), which was linked to reduced constitutive activation of NFkB and STAT3. Our data imply that Syndecan-1 depletion reduces the SP and CD44+/CD24-/low phenotype of MDA-MB-231 cells via interference with the NFkB and IL-6/STAT-3 signaling pathways (Figure 6). 

Although our results revealed that Syndecan-1-depleted cells displayed no alteration in the expression of Wnt-1, downregulation of LRP-6 may be responsible for reduced responsiveness to this pathway (Figure 6). A Frizzled receptor and either the LRP5 or LRP-6 co-receptor are essential for signal transduction through the canonical Wnt pathway during development and in disease [71]. LRP-6 is highly expressed in triple-negative breast cancers and its inhibition leads to attenuated Wnt activation, proliferation, and *in vivo* tumor growth [55,71]. Mounting evidence suggests that Wnt induces mammary tumor formation from stem or progenitor cells via LRP5/6-mediated activation of ß-catenin and mTOR pathways [72]. Intriguingly, Syndecan-1 deficient mice display resistance to mammary tumorigenesis due to a decreased Wnt-1-responsive progenitor cell population in mammary glands [36-38]. In line with our data in human breast cancer cell lines, these findings highlight that Syndecan-1 expression might potentiate Wnt signaling via regulation of LRP-6 coreceptor expression even in the absence of Wnt ligand overexpression [38]. Interestingly, the Wnt-pathway up-regulates STAT3 mRNA expression [73], and crosstalk of STAT3 with NFκB and the Wnt pathways apparently serves as a feed-forward loop [74]. Moreover, IL-6 maintains and augments the canonical Wnt signaling associated with low axin and high low density lipoprotein receptor-related protein (LRD), Dishevelled, and β-catenin levels in 3T3-L1 preadipocytes [75]. Overall, Syndecan-1 emerges as a key player intersecting and modulating these signaling pathways together, thus mediating breast CSC function.

In summary, we have demonstrated that a reduction of Syndecan-1 expression reduces breast CSC pools both in the aggressive triple negative human MDA-MB-231 cell line and in more differentiated, hormone receptor positive MCF-7 cells. Reduced CSC stemness is associated with downregulation of LRP-6, and attenuation of the NFκB and IL-6/STAT3 signaling pathways in triple-negative cells. Interference with Syndecan-1-dependent signaling emerges as a potential supportive targeting strategy in order to inhibit CSC stemness, thus preventing relapse after a successful convential therapy. Finally, our study underscores the viability and importance of flow cytometric techniques in analyzing CSC function.

## Supporting Information

Figure S1 **qPCR analysis of the EMT markers ZEB2 and SNAI1 in Syndecan-1 and control siRNA transfected MDA-MB-231 and MCF-7 cells reveals no significant expression differences.** Data are shown as fold change of expression in Syndecan-1 siRNA treated compared to control siRNA treated cells (n=3, P>0.05 (n.s.)). qPCR was performed essentially as previously described [Ibrahim SA et al. Int J Cancer 131:E884-896] using the following primers: ZEB2: fw: TGGGCTAGTAGGCTGTGTCCA , rev: TCATCTTCAACCCTGAAACAGAGG; SNAI1: fw:CCTGTTTCCCGGGCAATTTA, rev: TTCTGGGAGACACATCGGTCA.
(PPT)

Figure S2 **Syndecan-1 siRNA knockdown reduces LRP-6 expression in membrane fractions of MCF-7 and MDA-MB-231 cells.** 2 x 10^5^ MDA-MB-231 or MCF-7 cells were plated in a 6-well plate, incubated overnight in normal growth medium, and transfected with control and Sdc-1 siRNA after 24h. Post 24h, the transfection media were replaced with growth media containing 10% FCS. For separation of subcellular cytosolic and membrane fractions, the cells were washed twice with ice-cold PBS and lysed in ice cold 100 μl fractionation lysis buffer/well containing proteases inhibitors. Cells were scraped with a cell scraper and collected in an Eppendorf tube. The cells were then disrupted by 6-7 cycles of freezing in liquid nitrogen and thawing at 37°C. The crude lysate was subjected to centrifugation at 100,000 ×g for 30 min at 4°C and the supernatant was collected as the cytosolic fraction. Pellets were resuspended, and membrane proteins were homogenized in 100 μl of lysis buffer containing 2% Triton X-114. The homogenate was centrifuged at 800 ×g for 10 min. The membrane fraction was separated by SDS-PAGE and immunoblotted probing for LRP-6 and TLR4 as a loading control.
(PPT)
