# Supplementary material for: Syndecan-1 (CD138) Modulates Triple-Negative Breast Cancer Stem Cell Properties via Regulation of LRP-6 and IL-6-Mediated STAT3 Signaling
Source: PLoS One. 2013 Dec 31;8(12):e85737. doi: 10.1371/journal.pone.0085737 (PMC3877388; doi:10.1371/journal.pone.0085737)
Supplement: Figure S1 — qPCR analysis of the EMT markers ZEB2 and SNAI1 in Syndecan-1 and control siRNA transfected MDA-MB-231 and MCF-7 cells reveals no significant expression differences. Data are shown as fold change of expression in Syndecan-1 siRNA treated compared to control siRNA treated cells (n=3, P>0.05 (n.s.)). qPCR was performed essentially as previously described [Ibrahim SA et al. Int J Cancer 131:E884-896] using the following primers: ZEB2: fw: TGGGCTAGTAGGCTGTGTCCA , rev: TCATCTTCAACCCTGAAACAGAGG; SNAI1: fw:CCTGTTTCCCGGGCAATTTA, rev: TTCTGGGAGACACATCGGTCA. (PPT) [file pone.0085737.s001.ppt]

## Slide 1
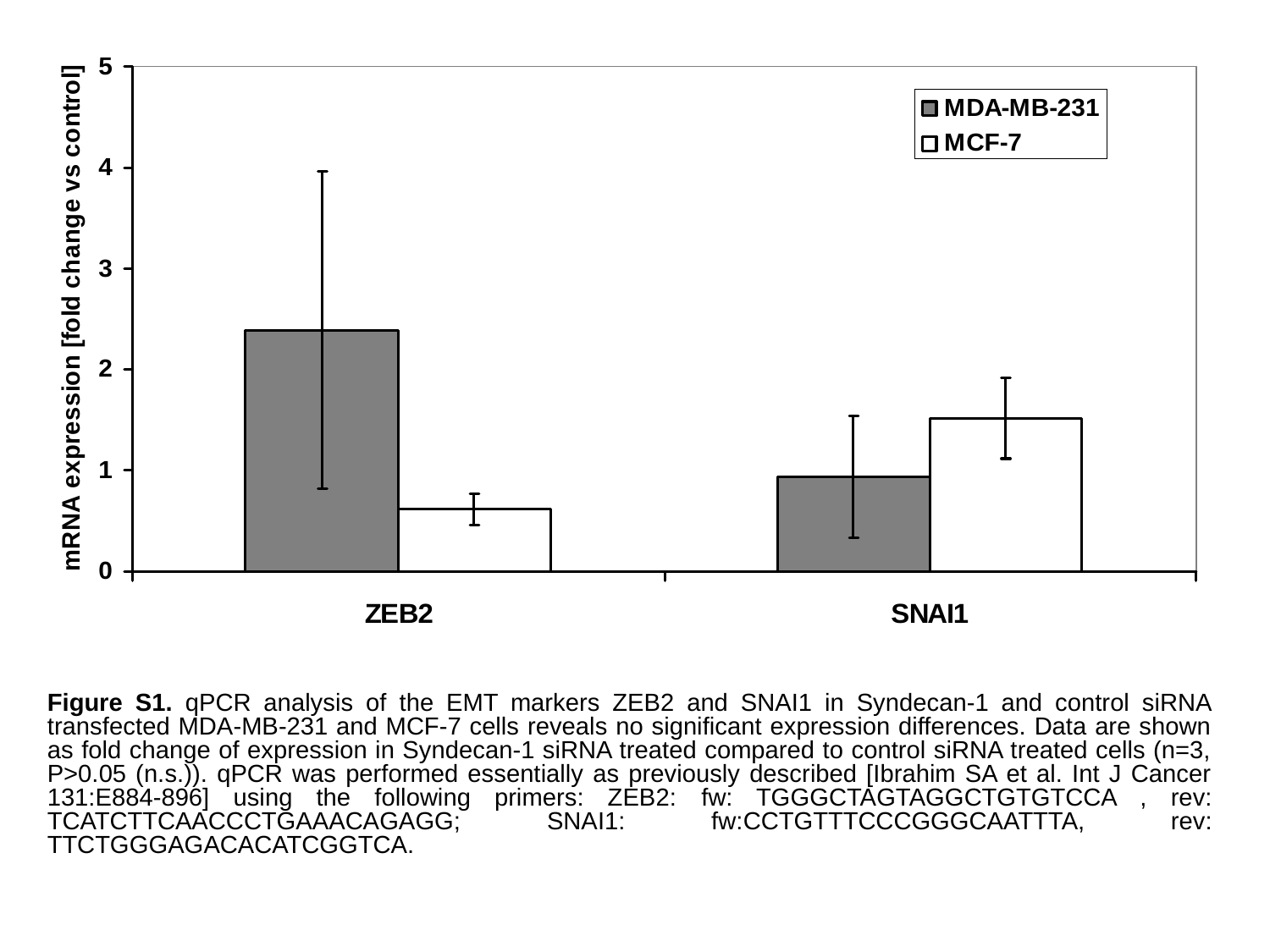

# Figure S1. qPCR analysis of the EMT markers ZEB2 and SNAI1 in Syndecan-1 and control siRNA transfected MDA-MB-231 and MCF-7 cells reveals no significant expression differences. Data are shown as fold change of expression in Syndecan-1 siRNA treated compared to control siRNA treated cells (n=3, P>0.05 (n.s.)). qPCR was performed essentially as previously described [Ibrahim SA et al. Int J Cancer 131:E884-896] using the following primers: ZEB2: fw: TGGGCTAGTAGGCTGTGTCCA , rev: TCATCTTCAACCCTGAAACAGAGG; SNAI1: fw:CCTGTTTCCCGGGCAATTTA, rev: TTCTGGGAGACACATCGGTCA.
