# Supplementary material for: Syndecan-1 (CD138) Modulates Triple-Negative Breast Cancer Stem Cell Properties via Regulation of LRP-6 and IL-6-Mediated STAT3 Signaling
Source: PLoS One. 2013 Dec 31;8(12):e85737. doi: 10.1371/journal.pone.0085737 (PMC3877388; doi:10.1371/journal.pone.0085737)
Supplement: Figure S2 — Syndecan-1 siRNA knockdown reduces LRP-6 expression in membrane fractions of MCF-7 and MDA-MB-231 cells. 2 x 10^5^ MDA-MB-231 or MCF-7 cells were plated in a 6-well plate, incubated overnight in normal growth medium, and transfected with control and Sdc-1 siRNA after 24h. Post 24h, the transfection media were replaced with growth media containing 10% FCS. For separation of subcellular cytosolic and membrane fractions, the cells were washed twice with ice-cold PBS and lysed in ice cold 100 μl fractionation lysis buffer/well containing proteases inhibitors. Cells were scraped with a cell scraper and collected in an Eppendorf tube. The cells were then disrupted by 6-7 cycles of freezing in liquid nitrogen and thawing at 37°C. The crude lysate was subjected to centrifugation at 100,000 ×g for 30 min at 4°C and the supernatant was collected as the cytosolic fraction. Pellets were resuspended, and membrane proteins were homogenized in 100 μl of lysis buffer containing 2% Triton X-114. The homogenate was centrifuged at 800 ×g for 10 min. The membrane fraction was separated by SDS-PAGE and immunoblotted probing for LRP-6 and TLR4 as a loading control. (PPT) [file pone.0085737.s002.ppt]

## Slide 1
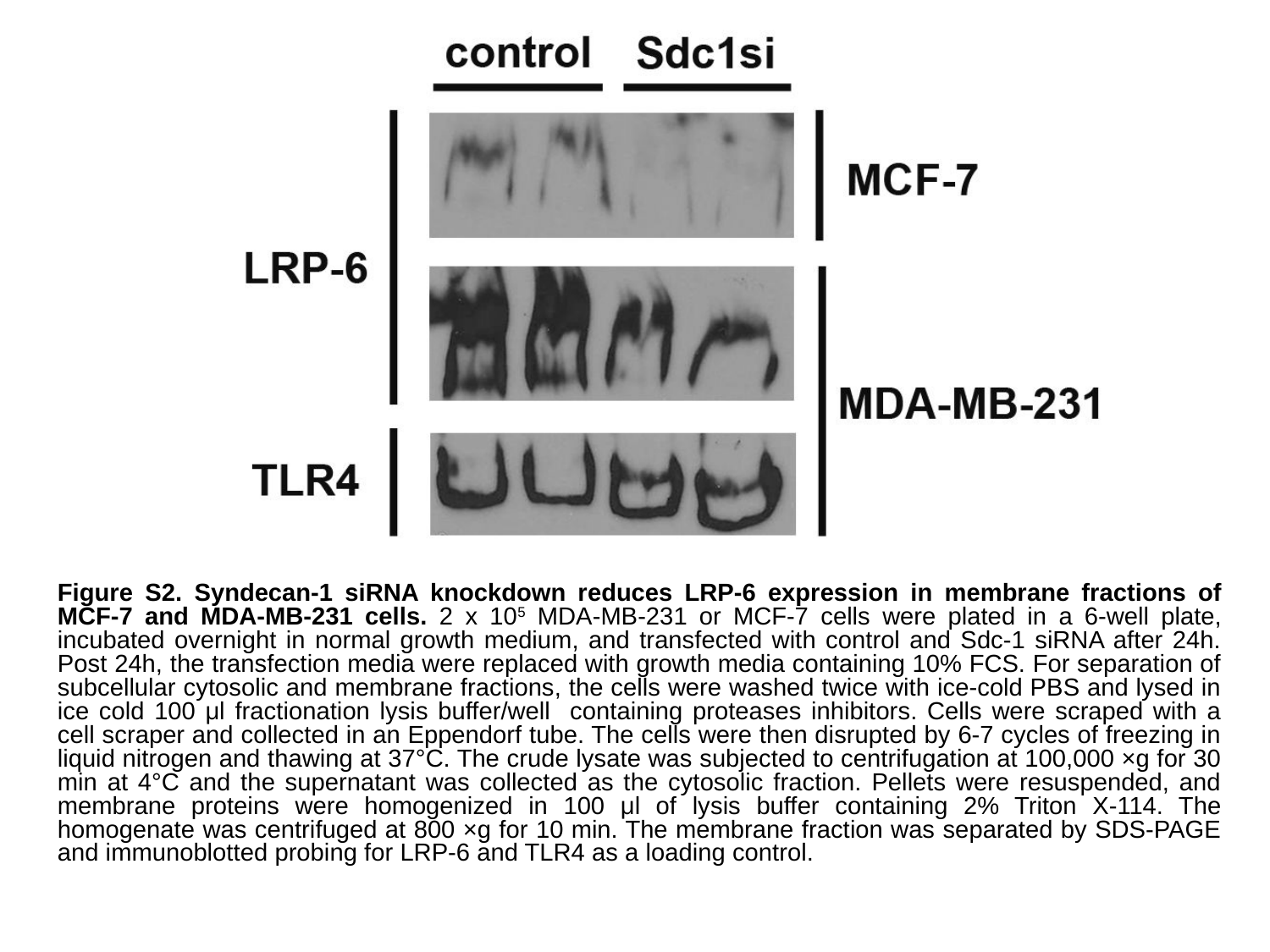

# Figure S2. Syndecan-1 siRNA knockdown reduces LRP-6 expression in membrane fractions of MCF-7 and MDA-MB-231 cells. 2 x 10^5^ MDA-MB-231 or MCF-7 cells were plated in a 6-well plate, incubated overnight in normal growth medium, and transfected with control and Sdc-1 siRNA after 24h. Post 24h, the transfection media were replaced with growth media containing 10% FCS. For separation of subcellular cytosolic and membrane fractions, the cells were washed twice with ice-cold PBS and lysed in ice cold 100 μl fractionation lysis buffer/well containing proteases inhibitors. Cells were scraped with a cell scraper and collected in an Eppendorf tube. The cells were then disrupted by 6-7 cycles of freezing in liquid nitrogen and thawing at 37°C. The crude lysate was subjected to centrifugation at 100,000 ×g for 30 min at 4°C and the supernatant was collected as the cytosolic fraction. Pellets were resuspended, and membrane proteins were homogenized in 100 μl of lysis buffer containing 2% Triton X-114. The homogenate was centrifuged at 800 ×g for 10 min. The membrane fraction was separated by SDS-PAGE and immunoblotted probing for LRP-6 and TLR4 as a loading control.
